# Added Value of Electronic Immunization Registries in Low- and Middle-Income Countries: Observational Case Study in Tanzania

**DOI:** 10.2196/32455

**Published:** 2022-01-21

**Authors:** Andrew M Secor, Hassan Mtenga, John Richard, Ngwegwe Bulula, Ellen Ferriss, Mansi Rathod, Tove K Ryman, Laurie Werner, Emily Carnahan

**Affiliations:** 1 PATH Seattle, WA United States; 2 PATH Dar es Salaam United Republic of Tanzania; 3 Immunization and Vaccine Development Program, Ministry of Health, Community Development, Gender, Elderly and Children Dar es Salaam United Republic of Tanzania; 4 Bill & Melinda Gates Foundation Seattle, WA United States

**Keywords:** immunization, immunization information system, electronic immunization registry, digital health, eHealth

## Abstract

**Background:**

There is growing interest and investment in electronic immunization registries (EIRs) in low- and middle-income countries. EIRs provide ready access to patient- and aggregate-level service delivery data that can be used to improve patient care, identify spatiotemporal trends in vaccination coverage and dropout, inform resource allocation and program operations, and target quality improvement measures. The Government of Tanzania introduced the Tanzania Immunization Registry (TImR) in 2017, and the system has since been rolled out in 3736 facilities in 15 regions.

**Objective:**

The aims of this study are to conceptualize the additional ways in which EIRs can add value to immunization programs (beyond measuring vaccine coverage) and assess the potential value-add using EIR data from Tanzania as a case study.

**Methods:**

This study comprised 2 sequential phases. First, a comprehensive list of ways EIRs can potentially add value to immunization programs was developed through stakeholder interviews. Second, the added value was evaluated using descriptive and regression analyses of TImR data for a prioritized subset of program needs.

**Results:**

The analysis areas prioritized through stakeholder interviews were population movement, missed opportunities for vaccination (MOVs), continuum of care, and continuous quality improvement. The included TImR data comprised 958,870 visits for 559,542 patients from 2359 health facilities. Our analyses revealed that few patients sought care outside their assigned facility (44,733/810,568, 5.52% of applicable visits); however, this varied by region; facility urbanicity, type, ownership, patient volume, and duration of TImR system use; density of facilities in the immediate area; and patient age. Analyses further showed that MOVs were highest among children aged <12 months (215,576/831,018, 25.94% of visits included an MOV and were applicable visits); however, there were few significant differences based on other individual or facility characteristics. Nearly half (133,337/294,464, 45.28%) of the children aged 12 to 35 months were fully vaccinated or had received all doses except measles-containing vaccine–1 of the 14-dose under-12-month schedule (ie, through measles-containing vaccine–1), and facility and patient characteristics associated with dropout varied by vaccine. The continuous quality improvement analysis showed that most quality issues (eg, MOVs) were concentrated in <10% of facilities, indicating the potential for EIRs to target quality improvement efforts.

**Conclusions:**

EIRs have the potential to add value to immunization stakeholders at all levels of the health system. Individual-level electronic data can enable new analyses to understand service delivery or care-seeking patterns, potential risk factors for underimmunization, and where challenges occur. However, to achieve this potential, country programs need to leverage and strengthen the capacity to collect, analyze, interpret, and act on the data. As EIRs are introduced and scaled in low- and middle-income countries, implementers and researchers should continue to share real-world examples and build an evidence base for how EIRs can add value to immunization programs, particularly for innovative uses.

## Introduction

### Background

With the increasing digitalization of health systems worldwide, there is growing interest and investment in electronic immunization registries (EIRs). EIRs are “confidential, computerized, population-based systems that collect and consolidate vaccination data from vaccination providers for better immunization strategies” [1]. EIRs are designed to provide a consolidated patient record to health care workers at the point of care to enable the delivery of the appropriate vaccines at the appropriate time. At the population level, EIRs can provide aggregate data on vaccination coverage to inform resource allocation and program operations. In this way, EIRs aim to improve the immunization delivery system to reach every child by supporting more effective, efficient, and data-driven care [2,3].

Vaccine coverage has historically been the primary metric for evaluating immunization programs. As an increasing number of low- and middle-income countries (LMICs) have begun implementing EIRs, vaccine coverage has been measured as a key outcome for assessing EIR effectiveness. Pre–post studies in Vietnam, Bangladesh, and Pakistan have demonstrated significant increases in child vaccination coverage after the introduction of EIRs that included SMS text message reminders and, in addition, in Pakistan, decision support systems [4-6].

In addition to improving vaccine coverage, other benefits of EIRs have been identified for individuals, immunization program performance and management, research, and population health [7,8]. For example, EIRs store patient vaccine history at the individual level, can help identify defaulters and reduce dropout rates at the program level, and provide data to support resource allocation and strategic planning at the population level [8]. Across levels, EIRs that capture individual-level data provide an opportunity to redefine traditional vaccine indicators and conduct more timely, granular analyses to support decision-making [9]. EIRs enable immunization programs to explore outcomes of interest beyond vaccine coverage, including longitudinal outcomes at the population and individual levels. As EIRs are costly to introduce and maintain, it is important for decision-makers to consider all possible benefits to justify the investment [10,11].

In some settings where EIRs are being considered or introduced, immunization coverage may already be high and, therefore, not an appropriate metric for EIR added value. The Early-Stage Digital Health Investment Tool was developed to assist ministries of health in determining their readiness to introduce a digital health tool, such as an EIR, by assessing the core building blocks of digital health [12]. In practice, country health systems with sufficient readiness are likely those that have already achieved relatively high vaccination coverage. In these contexts, improved vaccination coverage may not be the primary goal of introducing EIRs.

### Objective

The aims of this study are to (1) conceptualize additional ways that EIRs can add value to immunization programs and (2) assess the feasibility and potential value-add using Tanzania as a case study.

## Methods

### Overview

This study comprised 2 sequential phases. First, a comprehensive list of ways EIRs can potentially add value for immunization programs was developed through stakeholder interviews. Second, the added value was evaluated using Tanzania Immunization Registry (TImR) data for a prioritized subset of program needs.

### Phase 1

#### Conceptual Framework

A comprehensive list of common barriers that country immunization programs face in achieving coverage and equity goals was used to identify the ways in which EIRs can add value. The list was adapted from a July 2019 Gavi workshop on *Improving Data use in Immunization* in which approximately 40 participants from the Gavi Secretariat, core and extended partners, and country representatives identified and categorized barriers. For each common barrier, the study team (EC, TKR, and LW) identified ways that EIRs could help address the barrier based on their expertise and implementation experience.

#### Data Collection and Analysis

Stakeholder interviews were conducted to refine the framework of the immunization program barriers and potential EIR solutions. Stakeholders were purposively selected based on their expertise in research, policy, or implementation of EIRs. A total of 7 stakeholders participated in semistructured web-based interviews facilitated by the study team (EC) from November 2019 to January 2020. A total of 4 stakeholders were government officials from countries in Sub-Saharan Africa, identified through the BID (Better Immunization Data) Learning Network [13]. A total of 3 stakeholders were from international public health agencies, donors, or implementing organizations. Summary notes from the interviews were used to refine the conceptual framework. A follow-up web-based survey (using SurveyMonkey, Momentive, Inc) was sent to a wider group of EIR experts (including interviewees) in January 2020, asking respondents to prioritize topics from the conceptual framework for further analyses. Survey responses from 17 individuals were used, in conjunction with the interview data, to prioritize the 4 topics for phase 2 analyses.

### Phase 2

#### Setting

Data from Tanzania’s EIR were analyzed to illustrate how an EIR can add value to each of the prioritized topic areas. The Government of Tanzania partnered with the BID Initiative, funded by the Bill & Melinda Gates Foundation and launched in 2013, to design and implement a package of solutions to improve immunization data quality and use [14]. An EIR was an essential component of the solution package. EIR design began in 2014 and went through iterations culminating in TImR, which is built on the OpenIZ platform (now known as SanteDB, SanteSuite) [15]. The Government of Tanzania has led a staged rollout of TImR to facilities across districts and regions. As of December 31, 2020, TImR was rolled out to 3736 facilities across 15 of 25 regions in mainland Tanzania and included 1.6 million client records.

#### Data Sources

Immunization, facility, and patient data were extracted from the TImR system with permission from the Government of Tanzania. Data were deidentified after extraction, and all analyses were conducted using deidentified data. The development and implementation of the TImR system have been discussed in detail elsewhere [15-17]. Population density data were extracted at the ward level from WorldPop’s United Nations–adjusted GeoTIFFs at 100×100 km spatial resolution using Database of Global Administrative Areas (GADM) administrative shapefiles and matched to facilities based on facility geocodes [18,19]. Subject matter experts were consulted on the construction of analysis variables (eg, missed opportunities for vaccination [MOVs]). Data were processed and analyzed using Alteryx (version 2020.3; Alteryx, Inc), R (version 4.0.0; R Foundation for Statistical Computing), Tableau (version 2020.2; Tableau Software, Inc), and STATA (version 14.2; StataCorp LLC).

#### Ethics Approval

This study received nonresearch determination from the PATH. The Government of Tanzania and the PATH have data-sharing permissions in place that guided the use of TImR data for this study.

#### Data Restrictions

The analyses focused on services provided between 2017 and 2019 and the vaccine doses that were included in the official Tanzania vaccine schedule, specifically Bacillus Calmette–Guérin (BCG); oral polio vaccine (OPV); diphtheria, tetanus, pertussis, hepatitis B, and *Haemophilus influenzae* type b (Penta); pneumococcal conjugate vaccine (PCV); rotavirus (Rota); and measles-containing vaccine (Table 1). Data were further restricted to doses received while the TImR system was live (ie, doses logged in the system at the time of service or shortly afterward). Doses retroactively entered to complete patient medical records were included in the continuum of care analysis only. This analysis included back-entered doses for patients with at least one live TImR entry to capture the full picture of their vaccine history. A total of 3 regions (Mtwara, Rukwa, and Ruvuma) with <50 visits recorded in TImR by December 31, 2019, were excluded. In addition, patient IDs with >3 instances of a given vaccine dose (eg, OPV-1) were assumed to be dummy patient IDs used to log vaccinations provided during mass immunization campaigns and were excluded from the analysis. Patient IDs with up to 3 instances of a given dose were assumed to result from data entry errors.

**Table 1:** Tanzania vaccine schedule.^a^

Vaccine dose	Scheduled visit number	Age eligibility
BCG^b^-0 and OPV^c^-0	1	Birth or first contact
OPV-1, Penta^d^-1, PCV^e^-1, and Rota^f^-1	2	6 weeks
OPV-2, Penta-2, PCV-2, and Rota-2	3	10 weeks
OPV-3, Penta-3, and PCV-3	4	14 weeks
MCV^g^-1	5	9 months
MCV-2	6	18 months

^a^Inactivated polio vaccine immunization was excluded from our analyses as it was introduced partway through the analysis period. It would normally be received during visit 4 at the age of 14 weeks.

^b^BCG: Bacillus Calmette–Guérin.

^c^OPV: oral polio vaccine.

^d^Penta: diphtheria, tetanus, pertussis, hepatitis B, and *Haemophilus influenzae* type b.

^e^PCV: pneumococcal conjugate vaccine.

^f^Rota: rotavirus.

^g^MCV: measles-containing vaccine.

### Definitions

#### Outcomes

##### Missed Opportunities for Vaccination

MOVs were assessed at the visit level using the World Health Organization (WHO) definition: “any contact with health services by an individual (child or person of any age) who is eligible for vaccination (e.g., unvaccinated or partially vaccinated and free of contraindications to vaccination), which does not result in the person receiving one or more of the vaccine doses for which he or she is eligible” [20]. Dose eligibility was based on patient age, prior doses received, and time since the last dose of the vaccine sequence (if applicable). The MOV variable was constructed both as a binary (any MOV in a visit or not) and count (number of vaccine-specific MOVs per visit). Binary coding was used for the regression models, which motivated the use of logistic regression.

##### Vaccine Dropout

Vaccine-specific dropout for multidose vaccines was defined as receiving the first but not the last dose in the vaccine schedule (eg, receiving PCV-1 but not PCV-3). OPV dropout was defined as receiving OPV-0 or OPV-1 and not OPV-3; children who did not receive OPV-0 by the age of 2 were eligible for OPV-1 without receiving OPV-0; therefore, either vaccine can be treated as the starting dose. We also assessed dropout between birth doses and first follow-up visit, defined as receiving either of the birth doses (BCG or OPV-0) but none of the first follow-up visit doses (OPV-1, Penta-1, PCV-1, and Rota-1). Finally, we assessed overall dropout, which is defined as receiving at least one scheduled vaccine dose but not completing the full 14-dose schedule. The dropout variables were constructed as binaries (meeting criteria for dropout or not), motivating the use of logistic regression in the models.

##### Assigned Facility and Nonassigned Visits

Children are assigned a home facility when they are registered in the TImR system based on their preferences and where they plan to receive care. A nonassigned visit is a visit to any health facility other than the assigned visit. This variable was constructed as a binary variable (visit at home facility or not), motivating the use of logistic regression in the models.

#### Predictors

##### Dose Timeliness

A dose was considered timely if it was received within 7 days of the scheduled date (Table 1). In practice, in Tanzania, a child is generally considered a defaulter after 7 days past their scheduled date.

##### Urbanicity

A facility was designated as urban if the ward in which it is located had a population density of at least 500 persons per square km and rural if otherwise [21]. A patient was assumed to live in an urban area if their assigned facility, presumably near their residence, was designated as urban or rural, if otherwise.

##### Stockout

Facility stock use, including days of 0 stock, is recorded in TImR by facility and vaccine type. Vaccine-specific stockout was defined as any period in which the stock balance for a given vaccine was zero. A composite indicator was also constructed for the proportion of days with a stockout, with the number of days with a stockout for a primary vaccine (BCG, OPV, PCV, Penta, Rota, or measles-containing vaccine [MCV]) as the numerator and the number of days with facility stock data in the TImR system for each primary vaccine as the denominator.

##### Age

Age was defined in two ways: static age at the time of data extraction (December 31, 2019) and age at the time of a given visit. The 2 age variables were coded into 1-year categories up to the age of 5 years (ie, <12 months, 12-23 months, 24-35 months, 36-47 months, and 48-59 months), which is the upper limit of standard eligibility for most of the vaccines of interest.

### Regression Models

For all analyses, we used mixed-effects logistic regression to assess the factors associated with the various outcomes. In all models, relevant patient and facility characteristics were included as fixed effects, and nested random intercepts for region, district, and facility ID were used to account for clustering.

## Results

### Phase 1: EIR Added Value

Textbox 1 lists ways that EIRs can help address common barriers faced by immunization programs in achieving coverage and equity. EIRs can add value through existing functionalities (eg, the ability to identify underimmunized children) and through functionalities that may not be a core component of existing systems (eg, the ability to serve as a platform for remote, virtual supportive supervision).

On the basis of stakeholder input, 4 topics were prioritized for phase 2 analyses:

1. Denominators and population movements, including patient movement between facilities or geographic areas for care
2. MOVs, including their frequency and any associated characteristics
3. Continuum of care, including which children drop out and when in the vaccination schedule
4. Continuous quality improvement (CQI), including trends or outliers in data quality or service delivery, to inform targeted quality improvement efforts

The remainder of this section provides an overview of the TImR data and then provides results on each of the 4 priority topic areas to illustrate how EIR data can be used to better understand denominators and population movement, MOVs, continuum of care, and CQI.

**Immunization barriers and potential electronic immunization registry–based solutions.**

**Lack of understanding about what drives immunization demand**

- EIR data can identify un- or underimmunized children and explore drivers of their vaccination status (eg, geography, demographic characteristics, and facility type).
- EIR data can be used to analyze at what point children drop out of the continuum of care.
- EIRs can have embedded decision support to guide health workers in delivering tailored messages or services to increase acceptance and uptake.

**Overly complex processes**

- EIRs can be designed to streamline data capture and reduce the burden of data entry.
- EIRs can be designed to meet decision-making needs for end users.

**Skill level and availability of human resources**

- Access to data through EIRs can empower and motivate users and strengthen agency.
- If EIRs are designed with individual health worker log-ins, EIRs can track human resources based on active health worker profiles.
- EIR data can identify error rates of individual health workers and link them to additional training or supportive supervision.
- EIRs can have embedded training resources or capacity assessments.
- EIR data can be used to forecast service delivery needs by facility or district to optimize the distribution of human resources and session times.

**Geographic and social barriers to access**

- EIR data can identify un- or underimmunized children to explore whether they are concentrated in certain geographic areas and if they have shared demographic characteristics to inform targeted outreach.
- EIRs can track an individual’s vaccinations across public and private sector facilities.

**Microplanning challenges**

- EIRs can capture more accurate, timely, and complete denominators to inform microplanning.
- EIR data can be used to understand population movement or health-seeking behaviors to inform microplanning (eg, how common it is for children to move between multiple facilities).

**Inadequate introduction of new vaccines**

- EIR data on current vaccine delivery can be used to forecast the necessary stock and human resources to introduce new vaccines.

**Inadequate governance structures and capacities**

- The process of designing and introducing an EIR can help clarify and document governance structures related to immunization data.
- EIR data can provide more accurate denominator estimates to inform costing and budgeting for the EPI.

**A lack of resilience in leadership**

- EIRs can encourage continuous quality improvement by highlighting trends, outliers, or patterns that may require adaptive management.
- EIRs provide more timely, detailed data compared with traditional paper-based reporting, which enables timely, responsive action from leaders.
- EIRs can provide a platform for remote, web-based supportive supervision.

**Gaps in information systems**

- EIRs can show which facilities are entering data or not and factors associated with reporting.
- EIRs can be designed to mimic health worker workflows to streamline data collection and reporting practices.

**Poor quality of stock data from health facilities**

- EIR service delivery data can be triangulated to see how consistent it is with vaccine stock data and to forecast stock needs.
- EIR service delivery data can be used to inform decisions about vial size (eg, whether smaller vial sizes are needed in some areas to reduce waste).

**Poor quality of service delivery**

- EIRs can identify service delivery patterns to optimize health worker allocation and session timing to match demand.
- EIRs that capture check-in time and vaccination time can calculate patient wait times.
- EIRs can identify missed opportunities for vaccination.
- EIRs can include stock reorder alerts to reduce stockout frequency.

**Vaccine safety and effectiveness**

- EIR data triangulated with patient-level data on adverse events following immunization or surveillance data can answer questions about the effectiveness of vaccines given at different times.

### Phase 2: Tanzania Case Study

The sample size for the individual analyses varied because of differing inclusion criteria and missing data. In full, our sample comprised 2,444,803 vaccine doses over 958,870 visits for 559,542 patients. These visits occurred in 2359 health facilities covering 57 districts in 10 regions. The median (IQR) number of provided doses per facility per month was 40 (9-123), and the median number of visits was 17 (4-49). Table 2 provides participant demographics and facility characteristics.

**Table 2:** Patient and facility characteristics.^a^

Level and covariate				Number of visits, n (%)		Number of patients, n (%)		Number of facilities, n (%)
**Patient**								
	**Sex**							
		Female	472,782 (49.35)		275,605 (49.31)		N/A^b^	
		Male	485,195 (50.65)		283,361 (50.69)		N/A	
	**Age (as of December 31, 2019)**							
		<12 months	235,387 (24.55)		153,857 (21.61)		N/A	
		12-23 months	300,948 (31.39)		183,618 (25.8)		N/A	
		24-35 months	300,646 (31.35)		143,976 (20.23)		N/A	
		36-47 months	106,673 (11.12)		64,360 (9.04)		N/A	
		48-59 months	13,389 (1.4)		12,153 (1.71)		N/A	
		≥5 years	1,828 (0.19)		153,857 (21.61)		N/A	
	**Age (at time of visit; months)**							
		<12	833,349 (86.91)		N/A		N/A	
		12-23	111,259 (11.6)		N/A		N/A	
		24-35	10,138 (1.06)		N/A		N/A	
		36-47	2811 (0.29)		N/A		N/A	
		48-59	1283 (0.13)		N/A		N/A	
	**Urbanicity (of patient)**							
		Rural	624,726 (66.2)		365,459 (66.38)		N/A	
		Urban	318,972 (33.8)		185,106 (33.62)		N/A	
**Facility**								
	**Facility type**							
		Dispensary	343,525 (60.39)		1,953 (82.79)		343,525 (60.39)	
		Health center	152,496 (26.81)		311 (13.18)		152,496 (26.81)	
		Hospital	72,786 (12.8)		95 (4.03)		72,786 (12.8)	
	**Urbanicity (of facility)**							
		Rural	621,375 (65.78)		364,817 (65.67)		1873 (81.01)	
		Urban	323,284 (34.22)		190,689 (34.33)		439 (18.99)	
	**TImR^c^ use duration (as of December 31, 2019)**							
		0-5 months	5038 (0.53)		N/A		104 (4.45)	
		6-11 months	282,993 (29.63)		N/A		1041 (44.56)	
		1 year	183,826 (19.25)		N/A		625 (26.76)	
		≥2 years	483,201 (50.59)		N/A		566 (24.23)	
	**Region**							
		Arusha	270,099 (28.25)		112,963 (20.22)		271 (11.56)	
		Dar es Salaam	3062 (0.32)		2726 (0.49)		48 (2.05)	
		Dodoma	96,059 (10.05)		61,033 (10.93)		321 (13.69)	
		Geita	25,807 (2.7)		21,152 (3.79)		123 (5.25)	
		Kilimanjaro	93,231 (9.75)		52,367 (9.37)		294 (12.54)	
		Lindi	12,532 (1.31)		10,101 (1.81)		162 (6.91)	
		Morogoro	86,815 (9.08)		61,697 (11.04)		298 (12.71)	
		Mwanza	152,914 (15.99)		113,953 (20.4)		318 (13.56)	
		Njombe	11,469 (1.2)		9645 (1.73)		184 (7.85)	
		Tanga	204,246 (21.36)		113,002 (20.23)		326 (13.9)	

^a^Some categories will add up to more or less than the total number of visits, patients, or facilities because of missing data or patients having repeat visits or visits at multiple facilities.

^b^N/A: not applicable.

^c^TImR: Tanzania Immunization Registry.

### Denominators and Population Movement

#### Overview

**Figure 1:**
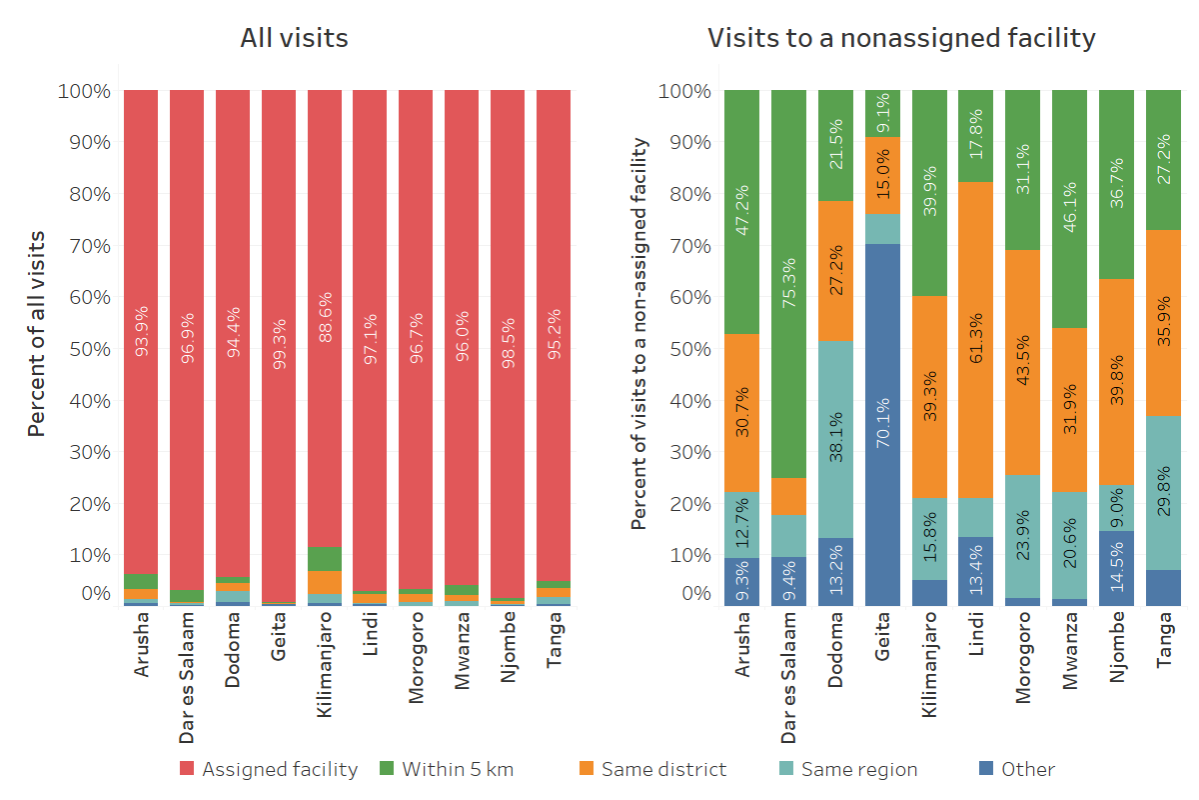
Visits to assigned and nonassigned facilities.

This analysis explored population movement, that is, care seeking at alternative (nonassigned) facilities, which affects the accuracy of facility denominators. Of 810,568 total visits, 765,835 (94.48%) were at a child’s assigned facility, 15,575 (1.92%) were at a nonassigned facility within 5 km of the child’s assigned facility, 14,147 (1.82%) at facilities located >5 km from the assigned facility but within the same district, 12,267 (1.51%) in a different district within the same region, and 2926 (0.36%) in a different region. Figure 1 summarizes attendance by region for all visits and visits to nonassigned facilities. Although children were similarly likely to seek care at their assigned facility across regions, patterns of care seeking to nonassigned facilities varied. For example, of visits to nonassigned facilities, children in Dar Es Salaam region were most likely to seek care within 5 km (64/85, 75%), whereas children in Geita were most likely to seek care outside of the region (131/187, 70.1%).

Table 3 explores visits at assigned and nonassigned facilities based on patient and assigned facility characteristics. There was little variation in the likelihood of a visit being at a nonassigned facility based on patient sex, assigned facility ownership, or assigned facility type. As expected, patients assigned to urban facilities and patients whose assigned facility had a higher number of facilities within 5 km were more likely to visit nonassigned facilities. Older children were more likely to visit nonassigned facilities; however, this could be an artifact of older children having more visits (and thus more opportunities to visit other facilities) or being more likely to have moved since being entered into the TImR system (ie, no longer residing near their assigned facility). Patients assigned to facilities newer to the TImR system were less likely to visit nonassigned facilities.

**Table 3:** Visits to assigned and nonassigned facilities by patient and assigned facility characteristics (N=810,568).

Covariate		Total visits, n	At nonassigned facility, n (%)	At assigned facility, n (%)
**Sex**				
	Female	400,507	22,028 (5.5)	378,479 (94.5)
	Male	409,164	22,504 (5.5)	386,660 (94.5)
**Age at time of visit (months)**				
	<12	232,653	10,935 (4.7)	221,718 (95.3)
	12-23	296,680	16,317 (5.5)	280,363 (94.5)
	24-35	220,613	12,796 (5.8)	207,817 (94.2)
	36-47	53,190	3989 (7.5)	49,201 (92.5)
	48-59	5908	520 (8.8)	5388 (91.2)
**Assigned facility type**				
	Dispensary	490,965	25,530 (5.2)	465,435 (94.8)
	Health center	223,344	13,401 (6.0)	209,943 (94.0)
	Hospital	96,259	5679 (5.9)	90,580 (94.1)
**Assigned facility urbanicity**				
	Rural	598,804	25,150 (4.2)	573,654 (95.8)
	Urban	205,070	19,482 (9.5)	185,588 (90.5)
**Assigned facility ownership**				
	Private	148,088	9922 (6.7)	138,166 (93.3)
	Public	655,786	34,101 (5.2)	621,685 (94.8)
**Assigned facility TImR^a^ duration (at time of visit)**				
	0-5 months	461,325	20,760 (4.5)	440,565 (95.5)
	6-11 months	196,127	14,513 (7.4)	181,614 (92.6)
	1 year	133,746	8693 (6.5)	125,053 (93.5)
	≥2 years	19,370	697 (3.6)	18,673 (96.4)
**Number of facilities within 5 km of assigned facility**				
	0	277,803	9167 (3.3)	268,636 (96.7)
	1	157,322	7551 (4.8)	149,771 (95.2)
	2-5	171,457	13,888 (8.1)	157,569 (91.9)
	>5	192,290	20,767 (10.8)	171,523 (89.2)

^a^TImR: Tanzania Immunization Registry.

#### Spatial Variation in Assigned Facility Attendance

**Figure 2:**
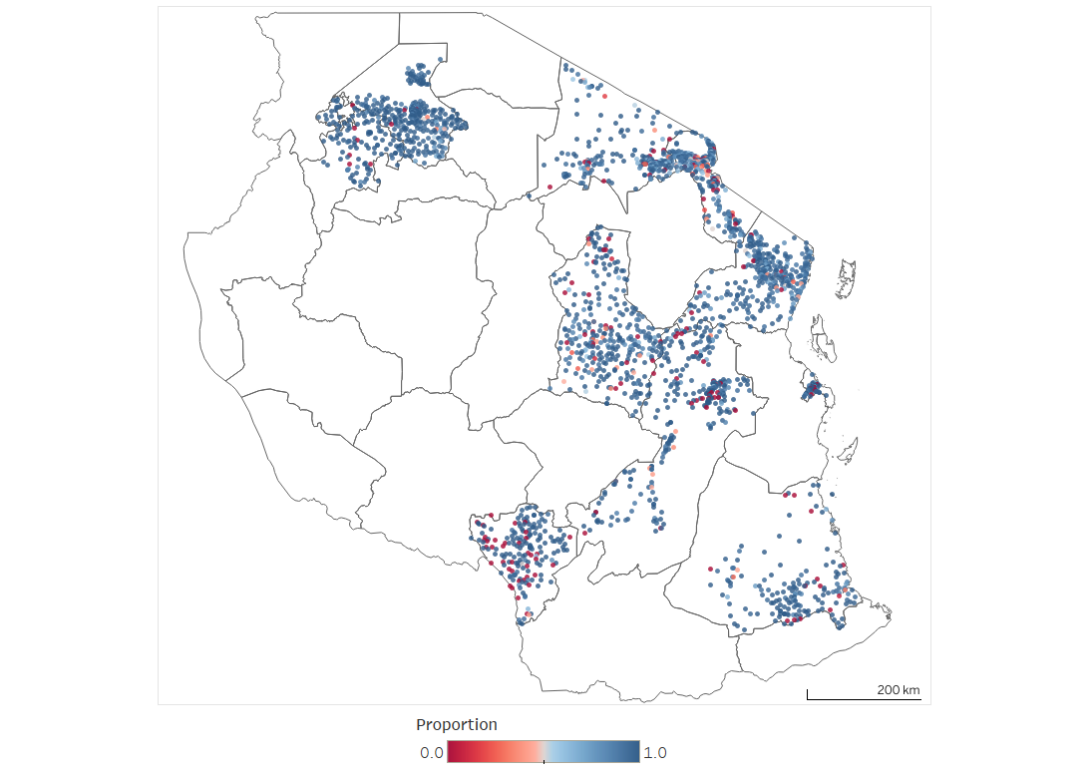
Proportion of visits at assigned facilities by facility geocode.

Figure 2 shows the proportion of all visits by children assigned to a given facility that occurred at the assigned facility. Facilities with low attendance appeared to cluster in northern Kilimanjaro, southeastern Arusha, southeastern and urban Mwanza, and coastal and central Tanga.

#### Model Results

Table 4 shows results from the logistic regression model with a given visit to a nonassigned facility as the outcome of interest. Children assigned to public facilities and health centers or hospitals, facilities with a longer duration of TImR use, and facilities in areas with higher health facility density were significantly more likely to visit a nonassigned facility. Children attending facilities with a greater number of recorded visits were significantly less likely to visit a nonassigned facility. Interestingly, the relationship with age was no longer monotonic after adjusting for other covariates. As compared with children aged ≤12 months, children aged 1 to 2 years were less likely to visit a nonassigned facility, whereas those aged 3 to 4 years were more likely.

**Table 4:** Population movement regression model results.

Covariate			Unadjusted model				Adjusted model		
			OR^a^ (95% CI)		*P* value		aOR^b^ (95% CI)		*P* value
**Sex**									
	Female	Reference		N/A^c^		Reference		N/A	
	Male	0.98 (0.96-1.00)		.08		0.99 (0.97-1.01)		.21	
**Age (months)**									
	<12	Reference		N/A		Reference		N/A	
	12-23	0.71 (0.69-0.74)		<.001		0.79 (0.76-0.82)		<.001	
	24-35	0.64 (0.61-0.66)		<.001		0.87 (0.83-0.91)		<.001	
	36-47	0.85 (0.81-0.89)		<.001		1.30 (1.23-1.37)		<.001	
	48-59	1.20 (1.08-1.33)		<.001		1.89 (1.69-2.11)		<.001	
**Assigned facility urbanicity**									
	Rural	Reference		N/A		Reference		N/A	
	Urban	1.42 (1.11-1.82)		.01		0.93 (0.74-1.18)		.56	
**Assigned facility ownership**									
	Private	Reference		N/A		Reference		N/A	
	Public	N/A		N/A		1.37 (1.13-1.66)		.001	
**Assigned facility type**									
	Dispensary	Reference		N/A		Reference		N/A	
	Health center	1.16 (0.94-1.43)		.17		1.71 (1.41-2.07)		<.001	
	Hospital	1.53 (1.07-2.21)		.02		2.13 (1.52-2.98)		<.001	
Assigned facility stockout (% of days)			1.00 (1.00-1.01)		.48		1.00 (0.99-1.01)		.94
Total assigned visits (log)			0.28 (0.26-0.32)		<.001		0.24 (0.21-0.27)		<.001
**Assigned facility TImR^d^ duration**									
	0-5 months	Reference		N/A		Reference		N/A	
	6-11 months	2.40 (1.17-4.94)		.02		1.55 (1.44-1.68)		<.001	
	12-23 months	2.84 (1.17-6.88)		.02		7.29 (6.75-7.87)		<.001	
	≥2 years	2.15 (0.88-5.28)		.09		8.15 (7.48-8.89)		<.001	
**Number of facilities within 5 km of assigned facility**									
	0	Reference		N/A		Reference		N/A	
	1	1.62 (1.50-1.76)		<.001		2.03 (1.97-2.09)		<.001	
	2-5	8.38 (7.75-9.05)		<.001		2.06 (1.98-2.15)		<.001	
	>5	9.55 (8.76-10.40)		<.001		1.48 (1.35-1.64)		<.001	

^a^OR: odds ratio.

^b^aOR: adjusted odds ratio.

^c^N/A: not applicable.

^d^TImR: Tanzania Immunization Registry.

### Missed Opportunities for Vaccination

#### Overview

MOVs, where the patient did not receive at least one vaccine for which they were eligible, were observed in 23.69% (226,525/956,195) of visits. Although we found little variation in the likelihood of an MOV based on sex, there was notable heterogeneity across age groups, facility urbanicity, facility type, and duration of TImR use at the facility (Table 5). The higher likelihood of an MOV among younger patients may be an artifact of the higher number of scheduled doses in the first year of life, and therefore, greater opportunity for missed doses.

**Table 5:** Visits with missed opportunities for vaccination (MOVs) by vaccine type and patient and facility characteristics.^a^

Covariate		Number of visits	Visits with an MOV by vaccine, n (%)						
			Any vaccine	Penta^b^	OPV^c^	BCG^d^	MCV^e^	Rota^f^	PCV^g^
Overall		956,195	226,525 (23.69)	60,364 (6.31)	58,040 (6.07)	54,924 (5.74)	5781 (0.60)	95,651 (10.00)	63,684 (6.66)
**Sex**									
	Female	471,406	111,636 (23.68)	29,692 (6.30)	28,570 (6.06)	27,234 (5.78)	2794 (0.59)	47,011 (9.97)	31,358 (6.65)
	Male	483,896	114,582 (23.68)	30,594 (6.32)	29,409 (6.08)	27,583 (5.70)	2953 (0.61)	48,488 (10.02)	32,236 (6.66)
**Age group (months)**									
	<12	831,018	215,576 (25.94)	55,453 (6.67)	53,492 (6.44)	51,461 (6.19)	2080 (0.25)	95,651 (11.51)	58,627 (7.05)
	12-23	110,968	9298 (8.38)	4376 (3.94)	4027 (3.63)	2913 (2.63)	2973 (2.68)	—^h^	4492 (4.05)
	24-35	10,123	1239 (12.24)	374 (3.69)	350 (3.46)	354 (3.5)	609 (6.02)	—	417 (4.12)
	36-47	2805	293 (10.45)	114 (4.06)	125 (4.46)	145 (5.17)	83 (2.96)	—	104 (3.71)
	48-59	1281	119 (9.29)	47 (3.67)	46 (3.59)	51 (3.98)	36 (2.81)	—	44 (3.43)
**Visited facility type**									
	Dispensary	563,186	138,714 (24.63)	38,917 (6.91)	34,295 (6.09)	30,271 (5.37)	3646 (0.65)	61,852 (10.98)	43,948 (7.80)
	Health center	270,290	58,821 (21.76)	14,359 (5.31)	16,463 (6.09)	15,233 (5.64)	1465 (0.54)	22,830 (8.45)	14,035 (5.19)
	Hospital	122,719	28,990 (23.62)	7088 (5.78)	7282 (5.93)	9420 (7.68)	670 (0.55)	10,969 (8.94)	5701 (4.65)
**Visited facility urbanicity**									
	Rural	620,214	156,768 (25.28)	41,729 (6.73)	41,956 (6.76)	35,968 (5.8)	4178 (0.67)	67,467 (10.88)	47,301 (7.63)
	Urban	322,199	65,247 (20.25)	16,588 (5.15)	14,540 (4.51)	18,082 (5.61)	1540 (0.48)	26,318 (8.17)	15,165 (4.71)
**Visited facility TImR^i^ duration (at time of visit)**									
	0-5 months	600,234	130,595 (21.76)	23,894 (3.98)	37,593 (6.26)	36,829 (6.14)	2340 (0.39)	53,416 (8.90)	36,747 (6.12)
	6-11 months	197,188	50,634 (25.68)	18,569 (9.42)	11,582 (5.87)	10,329 (5.24)	1321 (0.67)	21,660 (10.98)	12,598 (6.39)
	1 year	135,342	37,630 (27.8)	15,231 (11.25)	7277 (5.38)	5864 (4.33)	1827 (1.35)	17,584 (12.99)	11,410 (8.43)
	≥2 years	19,705	6576 (33.37)	2430 (12.33)	1140 (5.79)	1188 (6.03)	248 (1.26)	2871 (14.57)	2641 (13.40)
**Visited facility stockout (% days)**									
	<10%	666,531	155,134 (23.27)	40,379 (6.06)	41,754 (6.26)	34,730 (5.21)	3990 (0.60)	66,733 (10.01)	46,524 (6.98)
	10%-19%	153,392	36,516 (23.81)	9888 (6.45)	8543 (5.57)	10,200 (6.65)	960 (0.63)	15,191 (9.90)	8672 (5.65)
	20%-29%	77,730	21,007 (27.03)	6297 (8.10)	4226 (5.44)	7103 (9.14)	490 (0.63)	7957 (10.24)	4291 (5.52)
	≥30%	54,072	12,203 (22.57)	2998 (5.54)	3031 (5.61)	2683 (4.96)	306 (0.57)	5090 (9.41)	3552 (6.57)

^a^Vaccine-specific percentages do not add up to the total missed opportunity for vaccination (MOV) percentage as patients can have MOVs for multiple vaccine types in a single visit.

^b^Penta: diphtheria, tetanus, pertussis, hepatitis B, and *Haemophilus influenzae* type b.

^c^OPV: oral polio vaccine.

^d^BCG: Bacillus Calmette–Guérin.

^e^MCV: measles-containing vaccine.

^f^Rota: rotavirus.

^g^PCV: pneumococcal conjugate vaccine.

^h^Children are not considered eligible for rotavirus immunization after the first year of life.

^i^TImR: Tanzania Immunization Registry.

Of the 557,674 children included in the analysis, 167,115 (29.97%) had ≥1 MOVs. The mean number of MOVs per child was 0.61 (SD 1.20). Among the 167,115 children with an MOV, 85,697 (51.28%) had ≥1 MOV (range 1-15). Of 338,439 recorded MOVs, rotavirus was the most likely to have an MOV (accounting for 28.26% of all MOVs; n=95,650), followed by PCV (18.82%, 63,682), Penta (17.84%, 60,363), OPV (17.15%, 58,039), BCG (16.23%, 54,924), and MCV (1.71%, 5781). The lower MOV proportion for MCV was likely because of fewer visits where children were age-eligible for MCV (aged at least 9 months).

#### MOV Reasons

The TImR system allows providers to indicate the reasons why a scheduled and eligible dose was not provided. However, the reason will only be noted if a dose is knowingly not given and thus is absent for doses for which providers did not recognize the patient’s eligibility. For eligible doses that the provider logged as missed, the data indicated the mechanisms behind MOVs.

Table 6 details MOV reasons by vaccine type. Of 338,439 recorded MOVs, 183,623 (54.26%) had a listed reason: 177,624 (52.48%) were because of facility stockout, 2474 (0.73%) because of medical contraindication, 3184 (0.94%) because of being *late* (generally meant to indicate that the child is too old to start the vaccine sequence), 178 (0.05%) because of guardian refusal, and 163 (0.05%) because of expired stock. These reasons varied by vaccine type, with roughly three-quarters of Penta and PCV MOVs because of stockout but less than half for BCG, MCV, and rotavirus MOVs. Rotavirus MOVs were more likely to result from medical contraindications (913/95,650, 0.95%) compared with MOVs of the other vaccine types, whereas MCV had the highest likelihood of being missed because of guardian refusal (15/5781, 0.26%).

**Table 6:** Reasons for missed opportunities for vaccination (MOVs).

Vaccine type	Number of recorded MOVs	MOV reason (MOVs for given vaccine type), n (%)					
		Stockout	Medical contraindication	Late	Refusal	Expired stock	No reason provided
Overall	338,439	177,624 (52.48)	2474 (0.73)	3184 (0.94)	178 (0.05)	163 (0.05)	154,816 (45.74)
Rota^a^	95,650	34,315 (35.88)	913 (0.95)	761 (0.8)	34 (0.04)	38 (0.04)	59,589 (62.3)
OPV^b^	58,039	37,056 (63.85)	296 (0.51)	1118 (1.93)	37 (0.06)	31 (0.05)	19,501 (33.6)
Penta^c^	60,363	46,133 (76.43)	309 (0.51)	558 (0.92)	36 (0.06)	62 (0.1)	13,265 (21.98)
PCV^d^	63,682	47,712 (74.92)	434 (0.68)	834 (1.31)	39 (0.06)	37 (0.06)	14,626 (22.97)
BCG^e^	54,924	24,430 (44.48)	126 (0.23)	513 (0.93)	40 (0.07)	11 (0.02)	29,804 (54.26)
MCV^f^	5781	2694 (46.6)	12 (0.21)	117 (2.02)	15 (0.26)	5 (0.09)	2938 (50.82)

^a^Rota: rotavirus.

^b^OPV: oral polio vaccine.

^c^Penta: diphtheria, tetanus, pertussis, hepatitis B, and *Haemophilus influenzae* type b.

^d^PCV: pneumococcal conjugate vaccine.

^e^BCG: Bacillus Calmette–Guérin.

^f^MCV: measles-containing vaccine.

#### Model Results

Results from the any-vaccine MOV and OPV-specific MOV models were selected as illustrative examples of interest and are shown in Table 7. Unadjusted results can be found in Multimedia Appendices 1 and 2. Age group and TImR duration were significantly associated with any MOV and OPV-specific MOVs. Compared with children aged <1 year, older children were substantially less likely to experience MOVs in both models. This may be because of the greater opportunity for MOVs at younger ages because of more scheduled doses in the first year of life. Interestingly, TImR use duration at the time of visit showed opposite directionality between the models, with longer TImR implementation associated with a higher likelihood of any MOV but lower likelihood of OPV-specific MOVs, suggesting that there may be different mechanisms leading to MOVs by vaccine type.

**Table 7:** Missed opportunity for vaccination (MOV) regression model results.

Covariate		Any MOV		OPV^a^ MOV	
		aOR^b^ (95% CI)	*P* value	aOR (95% CI)	*P* value
**Sex**					
	Female	Reference	N/A^c^	Reference	N/A
	Male	1.00 (0.99-1.01)	.90	1.00 (0.98-1.02)	.88
**Age (months)**					
	0-11	Reference	N/A	Reference	N/A
	12-23	0.19 (0.18-0.19)	<.001	0.41 (0.40-0.43)	<.001
	24-35	0.25 (0.23-0.26)	<.001	0.33 (0.29-0.37)	<.001
	36-47	0.19 (0.17-0.22)	<.001	0.40 (0.33-0.49)	<.001
	48-59	0.18 (0.15-0.22)	<.001	0.33 (0.24-0.46)	<.001
**Urbanicity**					
	Rural	Reference	N/A	Reference	N/A
	Urban	0.90 (0.75-1.08)	.25	0.96 (0.73-1.26)	.78
**Ownership**					
	Private	Reference	N/A	Reference	N/A
	Public	1.02 (0.88-1.18)	.85	0.89 (0.71-1.12)	.31
**Facility type**					
	Dispensary	Reference	N/A	Reference	N/A
	Health center	0.89 (0.77-1.03)	.11	1.13 (0.91-1.40)	.28
	Hospital	0.99 (0.76-1.28)	.91	0.92 (0.62-1.36)	.68
**Facility TImR^d^ duration (at time of visit)**					
	0-5 months	Reference	N/A	Reference	N/A
	6-11 months	1.61 (1.58-1.63)	<.001	0.90 (0.88-0.93)	<.001
	12-23 months	2.27 (2.22-2.31)	<.001	0.73 (0.71-0.76)	<.001
	≥2 years	3.15 (3.03-3.27)	<.001	0.67 (0.62-0.72)	<.001

^a^OPV: oral polio vaccine.

^b^aOR: adjusted odds ratio.

^c^N/A: not applicable.

^d^TImR: Tanzania Immunization Registry.

### Continuum of Care

This analysis explored the vaccine dropout. To ensure common eligibility for doses, this analysis was restricted to children aged 12 to 47 months at the end of 2019 and focused on the 14 doses scheduled for the first year of life (ie, through MCV-1; Table 1).

#### Immunization Coverage

**Figure 3:**
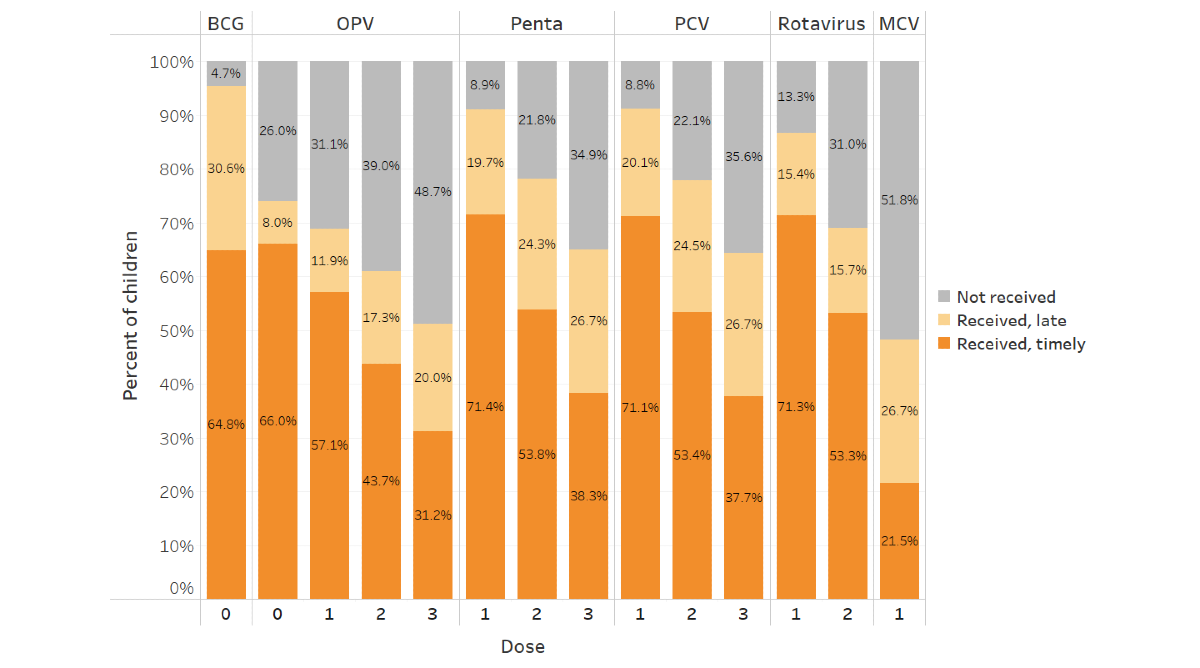
Vaccine coverage and dose timeliness. BCG: Bacillus Calmette–Guérin; MCV: measles-containing vaccine; OPV: oral polio vaccine; PCV: pneumococcal conjugate vaccine.

Overall, 93,619 (31.79%) of 294,464 children in our sample were fully immunized for doses scheduled in the first year of life (inclusive of OPV-0), with a further 39,718 (13.48%) receiving all scheduled doses, except for MCV-1. Figure 3 shows the doses received and timeliness by vaccine type and dose. As expected, there was a drop-off in coverage with later doses in each vaccine sequence. Timeliness also decreased monotonically with later doses in a sequence.

#### Vaccine Dropout

Table 8 details dropout rates across patient characteristics. For multidose vaccine-specific dropout, OPV had the highest rate (66,798/217796, 30.67%), followed by PCV (78,767/268,582, 29.33%), Penta (76,659/268,315, 28.57%), and Rota (52,086/255,337, 20.4%). Rotavirus may have had a lower dropout rate because there were only 2 doses in the sequence. There were common trends for all outcomes, such as older children and private facilities showing lower levels of dropout for all types of dropouts. However, some trends were outcome-/vaccine-specific, such as rural facilities showing higher levels of dropout for all vaccines except OPV. Most of these differences were marginal except for dropout by age group.

**Table 8:** Dropout by patient and facility characteristics.

Covariate			Children dropped out, n (%)										
			Penta^a^		OPV^b^		Rota^c^		PCV^d^		Birth or first		Overall dropout
Overall			76,659 (28.57)		66,798 (30.67)		52,086 (20.4)		78,767 (29.33)		16,414 (5.79)		194,765 (66.14)
**Sex**													
	Female	37,591 (28.47)		32,735 (30.6)		25,547 (20.31)		38,652 (29.23)		8073 (5.78)		95,793 (66.1)	
	Male	38,962 (28.66)		33,955 (30.69)		26,466 (20.45)		40,006 (29.4)		8262 (5.75)		98,633 (66.14)	
**Age group (months)**													
	12-23	51,057 (31.9)		46,743 (34.42)		34,951 (22.84)		52,795 (32.87)		12,159 (6.99)		124,599 (69.99)	
	24-35	25,602 (23.65)		20,055 (24.46)		17,135 (16.75)		25,972 (24.06)		4255 (3.88)		70,166 (60.26)	
**Assigned facility type**													
	Dispensary	46,833 (27.82)		38,737 (28.63)		32,216 (20.08)		48,415 (28.72)		8338 (4.72)		118,279 (64.57)	
	Health center	21,375 (29.52)		20,176 (33.41)		14,236 (20.62)		21,790 (30.06)		5161 (6.74)		54,369 (68.01)	
	Hospital	8451 (30.69)		7885 (35.68)		5634 (21.77)		8562 (31.15)		2915 (9.63)		22,117 (70.55)	
**Assigned facility urbanicity**													
	Rural	51,651 (28.88)		41,372 (30.23)		35,904 (21.19)		53,075 (29.68)		10,448 (5.54)		131,395 (67)	
	Urban	23,375 (27.46)		23,778 (30.94)		15,051 (18.44)		24,077 (28.25)		5711 (6.34)		59,872 (64.1)	
**Assigned facility ownership**													
	Private	14,514 (30.83)		11,976 (32.64)		9272 (20.78)		14,567 (30.89)		3072 (6.16)		35,856 (68.61)	
	Public	61,289 (28.03)		53,867 (30.13)		42,184 (20.26)		63,223 (28.9)		13,186 (5.71)		156,847 (65.54)	

^a^Penta: diphtheria, tetanus, pertussis, hepatitis B, and *Haemophilus influenzae* type b.

^b^OPV: oral polio vaccine.

^c^Rota: rotavirus.

^d^PCV: pneumococcal conjugate vaccine.

#### Immunization Typologies

To better understand vaccination profiles, we constructed immunization archetypes using all possible combinations of eligible scheduled doses. Patients were fit into these archetypes based on their immunization history. Table 9 shows the 10 most common archetypes by vaccine doses received. After being fully vaccinated and fully vaccinated except for MCV-1, the third most common typology was receiving all doses except for the OPV sequence (19,322/294,464, 6.56% of children), followed by dropping out between the second and third visits (13,270/294,464, 4.51%) or between the third and fourth visits (13,102/294,464, 4.45%), and receiving only the birth doses (BCG and OPV-0; 10,156/294,464, 3.45%).

**Table 9:** Immunization typologies (10 most common).

Vaccine and dose																				Children, n (%)
BCG^a^	OPV^b^					Penta^c^				PCV^d^				Rota^e^			MCV^f^			
0	0	1	2	3	1		2	3	1		2	3	1		2	1				
R^g^	R	R	R	R	R		R	R	R		R	R	R		R	R		93,619 (31.79)		
R	R	R	R	R	R		R	R	R		R	R	R		R	NR^g^		39,718 (13.49)		
R	NR	NR	NR	NR	R		R	R	R		R	R	R		R	R		19,322 (6.56)		
R	R	R	NR	NR	R		NR	NR	R		NR	NR	R		NR	NR		13,270 (4.51)		
R	R	R	R	N	R		R	NR	R		R	NR	R		R	NR		13,102 (4.45)		
R	R	NR	NR	NR	NR		NR	NR	NR		NR	NR	NR		NR	NR		10,156 (3.45)		
R	NR	NR	NR	NR	R		R	R	R		R	R	R		R	NR		10,064 (3.42)		
R	NR	NR	NR	NR	R		NR	NR	R		NR	NR	R		NR	NR		5587 (1.9)		
R	NR	NR	NR	NR	R		R	NR	R		R	NR	R		R	NR		3861 (1.31)		
R	R	R	R	NR	R		R	NR	R		R	NR	R		NR	NR		3842 (1.31)		

^a^BCG: Bacillus Calmette–Guérin.

^b^OPV: oral polio vaccine.

^c^Penta: diphtheria, tetanus, pertussis, hepatitis B, and *Haemophilus influenzae* type b.

^d^PCV: pneumococcal conjugate vaccine.

^e^Rota: rotavirus.

^f^MCV: measles-containing vaccine.

^g^"R" indicates a given dose was received, while "NR" indicates the dose was not received.

#### Visit Dropout

To understand dropout between different scheduled visits, we analyzed the proportion of children that had received any vaccine from each of the 5 scheduled touchpoints with the immunization system in the first year of life (Table 1). Overall, 96.29% (283,548/294,464) of children received at least one of the birth doses (BCG or OPV-0), 93.16% (274,314/294,464) received at least one of the visit 2 doses (OPV-1, PCV-1, Penta-1, or Rota-1), 80.98% (238,450/294,464) received at least one visit 3 dose, 67.52% (198,812/294,464) received at least one visit 4 dose, and 48.21% (141,948/294,464) received the visit 5 dose.

#### Model Results

The results from the Penta and overall dropout models were selected as illustrative examples of interest and are shown below. Unadjusted results can be found in Multimedia Appendices 3 and 4. As shown in Table 10, older age was significantly associated with a lower likelihood of both Penta and overall dropout (ie, starting but not finishing the 14-dose schedule). In the overall dropout model, urban facilities were associated with a significantly lower likelihood of overall dropout, and public facilities were associated with a higher likelihood. These trends were not observed in the Penta dropout model.

**Table 10:** Dropout regression model results.

Covariate			Penta dropout				Overall dropout		
			aOR^a^ (95% CI)		*P* value		aOR (95% CI)		*P* value
**Sex**									
	Female	Reference		N/A^b^		Reference		N/A	
	Male	1.02 (1.00-1.04)		.06		1.01 (0.99-1.03)		.21	
**Age (months)**									
	12-23	Reference		N/A		Reference		N/A	
	24-35	0.23 (0.22-0.23)		<.001		0.19 (0.19-0.19)		<.001	
**Assigned facility urbanicity**									
	Rural	Reference		N/A		Reference		N/A	
	Urban	0.86 (0.71-1.04)		.11		0.83 (0.70-0.99)		.03	
**Assigned facility ownership**									
	Private	Reference		N/A		Reference		N/A	
	Public	1.04 (0.90-1.22)		.57		1.15 (1.00-1.33)		.047	
**Assigned facility type**									
	Dispensary	Reference		N/A		Reference		N/A	
	Health center	0.95 (0.81-1.10)		.49		1.04 (0.91-1.22)		.58	
	Hospital	1.19 (0.91-1.54)		.20		1.27 (1.00-1.61)		.06	
Assigned facility stockout (% of days)			1.00 (1.00-1.01)		.12		1.00 (1.00-1.01)		.28

^a^aOR: adjusted odds ratio.

^b^N/A: not applicable.

### Continuous Quality Improvement

EIRs provide data for the rapid assessment of CQI improvement measures. These assessments can help improve service provision by identifying areas in need of targeted training or other quality improvement interventions. As shown in Table 11, 10% of facilities account for most of the issues, suggesting that targeted interventions to identified facilities could greatly improve care. These results use absolute numbers and, therefore, will be biased toward facilities with higher patient loads and longer TImR implementation durations. In practice, CQI analyses would likely be restricted to specific months or quarters, reducing any duration bias. Absolute figures can also offer greater efficiency by targeting CQI interventions to providers or facilities with the highest absolute number of issues.

**Table 11:** Continuous quality improvement.

All facilities (n=2345)	Issues accounted for, n (%)		
	Visits to a nonassigned facility^a^ (n=44,733)	Visits with an MOV^b^ (any vaccine; >n=226,525)	Children who have dropped out (full dropout; n=194,765)^a^
10% (n=134)	36,307 (81.16)	126,226 (55.72)	112,895 (57.96)
25% (n=586)	42,937 (95.99)	180,752 (79.79)	159,584 (81.94)
50% (n=1172)	44,715 (99.96)	215,989 (95.35)	188,281 (96.67)
75% (n=1758)	44,733 (100)	225,569 (99.58)	193,971 (99.59)

^a^Aggregated by child’s assigned facility.

^b^MOV: missed opportunity for vaccination.

## Discussion

### Principal Findings

EIRs can add value in multiple ways. Access to individual-level data that captures all touchpoints with the immunization program allows for new analyses that can benefit immunization programs, national and regional ministry staff, health care providers and administrators, funders, and other stakeholders [8,9,22]. Descriptive statistics can be used to rapidly monitor service provision and vaccination coverage or inform quality improvement efforts. Longitudinal and spatial analyses can be used to understand temporospatial changes in care and coverage. Risk factor analyses can be used to identify patient and facility characteristics associated with immunization issues (eg, dropout). These analyses can be targeted to the relevant stakeholder groups. For example, facility-level statistics for a given district can inform targeted supportive supervision, and national-level coverage trends can enable evidence-based policy development. EIRs also allow for more cost-effective and rapid synthesis of immunization data; many of these descriptive statistics and analyses would not be possible using the aggregate data available in the routine health information system or would require significant additional funding, time, and other resources for survey data collection [9]. The analyses presented in this study are intended to illustrate the types of insights that EIR data can provide to immunization programs.

### Denominators and Population Movement

Inaccurate population denominators are a common challenge for monitoring coverage, improving implementation, and informing planning, such as projecting vaccine stock and staffing needs. A recent scoping review of immunization data quality in LMICs found that denominators were often inaccurate, infrequently adjusted, and inconsistent between the district and national levels [23]. Population denominators are influenced by migration, urbanization, and refugee crises, among other population dynamics that can have large effects at the local level [24]. Population denominators are further complicated by children seeking care at different facilities over time. The Strategic Advisory Group of Experts working group on the quality and use of global immunization and surveillance data identified inaccurate denominators as a common challenge and noted the lack of guidance on how to improve the accuracy of denominators and track mobile populations [25].

EIRs greatly simplify tracking patients who seek care at multiple facilities, enabling a more nuanced understanding of population movement across both geography and time and allowing for more robust coverage estimates. The use of the TImR data allowed us to explore both the magnitude of and factors associated with seeking care at facilities other than the patient’s assigned, or *home*, facility. Our analysis revealed that a small subset of patients sought care outside their assigned facility (44,733/810,568, 5.52%); however, this varied by region; facility urbanicity, type, ownership, patient volume, and duration of TImR system use; density of facilities in the immediate area; and patient age. In addition, where patients seeking care varied by region, patients in some regions were more likely to travel to other districts and regions for care. For example, children who did not attend their assigned facility in Geita were most likely to attend a facility outside of the region, potentially because of population mobility associated with mining in the region. These insights can help inform resource allocation. EIRs also greatly simplify tracking patients who seek care at multiple facilities, decreasing the likelihood of missed or redundant doses. Although a small number of children in the Tanzania case study sought care outside their assigned facilities, some areas would have a much larger nomadic or mobile population. For example, in Cameroon, children born at home, immigrants, emigrants, and nomadic populations are not accurately accounted for when planning outreach vaccination sessions, which contributes to delaying or not vaccinating an estimated 30% to 70% of the population in some districts [26].

### Missed Opportunities for Vaccination

Identifying and avoiding MOVs is an important and cost-effective method for achieving greater vaccination coverage. The challenge is in identifying when, where, and among which children or facilities MOVs are experienced to address them. Integration of clinical decision support systems within EIRs can automate the determination of child dose eligibility and alert the provider, which has been shown to reduce MOVs for routine childhood immunizations [27,28]. In addition, by collating vaccination history with child and facility characteristics, EIRs naturally allow for exploration of MOVs across these characteristics and by different vaccines. Our analysis of the TImR data showed that MOVs were highest among children aged <12 months (as mentioned, potentially because of the higher number of scheduled doses in the first year of life); however, there were few significant differences by other individual or facility characteristics. Other studies of countries in Sub-Saharan Africa have identified additional demographic and socioeconomic characteristics associated with increased odds of MOVs, including high birth order, high number of under 5 children in the house, lack of maternal education, lack of media access, and household and neighborhood poverty [29,30]. Although these data were not captured in TImR, they could be captured by an EIR to enable new analyses and equity insights [8,31].

This information can be used by providers to identify children who may be at higher risk of experiencing an MOV. In addition, it can be used by managers to identify providers and facilities with higher rates of MOVs for supportive supervision or refresher training or identify areas with high rates of vaccine hesitancy for outreach campaigns. In addition, EIRs can provide insight into the mechanisms behind MOVs, such as stock issues and vaccine-specific hesitancy. Where data were available, stockouts were the primary reason for MOVs, whereas mechanisms such as vaccine hesitancy and medical contraindications were relatively rare. The TImR data also showed that rotavirus was the most likely to have an MOV, which may indicate that eligibility requirements should be reviewed or refresher training provided. For additional insights, analysis of EIR data can be complemented by other tools such as those included in the WHO MOV strategy toolkit [20].

### Continuum of Care

Identifying where in the vaccine schedule some children drop out and why they drop out is another key challenge for achieving high levels of vaccine coverage. Understanding which vaccine doses and child and facility characteristics are associated with failure to complete a vaccine sequence or the full vaccine schedule can help inform service provision, training, and quality improvement measures at the facility, regional, and national levels. In the TImR data, nearly half of children aged 12 to 35 months were fully vaccinated or had received all doses except MCV-1 of the 14-dose under-12-month schedule (ie, through MCV-1). Among children who did not complete the vaccine schedule, levels of dropout varied by vaccine. Facility characteristics associated with dropout also varied by vaccine; for example, assigned facility urbanicity was significantly associated with a lower likelihood of overall dropout (ie, starting but not finishing the 14-dose schedule) but not Penta-specific dropout, suggesting that the mechanisms behind dropout may vary by vaccine. Continuum of care analyses could be further expanded if the EIR data were linked to a birth registration system. In the Tanzania case study, 5.79% (16,414/283,548) of children dropped out between birth and the first immunization dose; however, this may be an underestimation if some children are not registered at birth. In countries with a strong civil registration and vital statistics system, linking the EIR to birth registration or an antenatal care registry could expand the continuum of care analysis. Using EIRs to explore immunization typologies can also provide insight into which vaccines and visits require greater care. For example, in the TImR data, 6.56% (19,322/294,464) of children were fully vaccinated through MCV-1 except for the 3 to 4 OPV doses, highlighting the need for greater research into barriers to OPV coverage.

### CQI Analysis

The CQI analysis showed that most issues (eg, MOVs) came from a minority of facilities. EIRs enable decision-makers at the national and subnational levels to quickly assess and identify providers, facilities, or geographic areas for targeted quality improvement measures, thereby improving the quality of care and increasing improvement in intervention effectiveness.

### Added Value of EIRs

These analyses were designed to show the potential of EIRs to allow for a more nuanced, rapid, and cost-effective evaluation of vaccine program data to facilitate data use for decision-making. For example, automated dashboards of key indicators (eg, vaccine-specific coverage, stockouts, and child dropout) can inform planning and clinical practice at the facility level without the need for on-site data analysis. Providers can also use EIRs to simplify the tracking of individual patients, particularly those seeking care at multiple facilities, to improve the quality of care and reduce issues such as MOVs [27,28]. The integration of EIRs with SMS text messaging services to automate appointment and outreach to children at risk of defaulting has been shown to reduce dropout rates for routine childhood immunizations [4]. At the district and regional levels, the evaluation of underperforming facilities can be used for targeted supportive supervision and supplemental training. At the national level, up-to-date data on geographic and spatial trends in vaccine coverage can be used to inform nationwide campaigns, resource allocation, or policy development.

Designed well, EIRs can democratize immunization data. However, they require the necessary support to function effectively. The Early-Stage Digital Health Investment Tool has identified 6 building blocks for effective digital health systems: human capacity, investments and funding, data capture and use, infrastructure, standards and interoperability, and governance and policy [12]. Strong building blocks can maximize the effectiveness of EIR systems; however, this can pose a challenge in some low- and middle-income settings where 1 or several of these building blocks may be lacking. The WHO estimates that 50% of low-income and 24% of lower-middle–income countries have strong institutional capacity or involvement in data analysis at the national ministry [22]. With technical capacity often centralized at the national level, these figures are even lower at the subnational level. Furthermore, 54% of low-income and 41% of lower-middle–income countries are rated nascent, limited, or moderate in their capacity to have data and evidence drive policy and planning [22]. Implementing robust and routinized data frameworks, including EIRs, can address gaps in data availability and provide mechanisms to harness the data to drive evidence-based policy and planning. Automation and tailoring of data output to specific end users (eg, facility-level indicator dashboards for providers) can simplify data analysis and interpretation. However, effective use of EIR data for decision-making will require health care workers and administrators at all levels to have the skills, motivation, and autonomy to understand and act on the data [16,17]. Leadership at the national and regional levels should prioritize capacity building to enable the health system to make use of EIR data [32].

EIR is a solution that aims to improve immunization program performance. The efficiency and impact of EIRs can be maximized by introducing them in combination with other interventions, such as capacity strengthening for data use, vaccine stock management systems, data governance frameworks, or SMS text messaging reminders for caregivers. Interventions that use multiple mechanisms to address various barriers to data use have been found to be more successful in achieving immunization data use and action [33].

### Limitations

The TImR results are intended to illustrate the ways EIRs can add value to immunization programs by providing actionable information for health care providers and managers. The results are not intended to be generalizable to Tanzania as a whole because of several data limitations. First, regions and districts implemented TImR at various points in time, meaning that some geographies are over- or underrepresented in the results. Second, and relatedly, only a subset of regions in Tanzania have introduced TImR; therefore, immunization services delivered outside the TImR coverage area are not captured in the results. Third, children who may live within the TImR coverage area but have not had a touchpoint with the immunization delivery system (also known as *zero dose children*) or were not registered at birth are not captured in the results. Fourth, this study did not assess data completeness, and any incomplete data (eg, providers not entering all immunizations into TImR) may limit the accuracy of the results. Fifth, prior studies of the TImR data have shown reduced system use over time, potentially biasing results toward facilities with greater capability to maintain reporting systems [16]. Sixth, as mentioned earlier, the analyses were limited to data captured in TImR. Although these data can be powerful for diagnosing issues, they do not capture all patient, facility, or geographic characteristics that may influence immunization delivery and can be limited in explaining trends. For example, MOVs may be underestimated as this study only captures MOVs during immunization visits and not during nonimmunization visits [34,35]. As noted earlier, some characteristics shown to be associated with MOVs were not captured in TImR. Triangulation with other data sources or targeted follow-up data collection can help answer the *why* questions. Finally, the lens used in this study was the assessment of the potential added value of EIRs. This study does not attempt to highlight the challenges associated with implementing or maintaining EIRs, although many such challenges have been identified elsewhere [1,3,8,9,17,36].

### Conclusions

EIRs have the potential to add substantial value to immunization stakeholders at all levels of the health system beyond measuring vaccine coverage. Individual-level data captured through EIRs can enable new analyses to understand immunization service delivery or care-seeking patterns, potential risk factors for underimmunization, and where challenges occur. Notably, most issues (eg, occurrence of MOVs, visits to a nonassigned facility, and number of defaulters) occur in a minority of facilities, highlighting the potential for EIRs to inform targeted quality improvement efforts. However, to achieve this potential, country programs need to leverage and strengthen their capacity for collecting, analyzing, and interpreting the data. Measures and analyses should be prioritized to match the needs and capabilities of the immunization program. Ideally, the prioritized measures should be integrated into routine systems to facilitate ongoing CQI efforts. As EIRs are introduced and scaled in LMICs, implementers and researchers should continue to share real-world examples and build an evidence base for how EIRs can add value to immunization programs, particularly for innovative uses.

## Appendix

Multimedia Appendix 1 Complete missed opportunity for vaccination (any vaccine) regression model results.

Multimedia Appendix 2 Complete missed opportunity for vaccination (oral polio vaccine) regression model results.

Multimedia Appendix 3 Complete dropout (Penta) regression model results.

Multimedia Appendix 4 Complete dropout (overall) regression model results.

## Abbreviations

BCG: Bacillus Calmette–Guérin
BID: Better Immunization Data
CQI: continuous quality improvement
EIR: electronic immunization registry
GADM: Database of Global Administrative Areas
LMIC: low- and middle-income country
MCV: measles-containing vaccine
MOV: missed opportunity for vaccination
OPV: oral polio vaccine
TImR: Tanzania Immunization Registry
WHO: World Health Organization

## References

[1] Danovaro-Holliday MC, Contreras MP, Pinto D, Molina-Aguilera IB, Miranda D, García O, Velandia-Gonzalez M (2019). Assessing electronic immunization registries: the Pan American Health Organization experience. Rev Panam Salud Publica.

[2] Freeman VA, DeFriese GH (2003). The challenge and potential of childhood immunization registries. Annu Rev Public Health.

[3] Namageyo-Funa A, Samuel A, Bloland P, Macneil A (2018). Considerations for the development and implementation of electronic immunization registries in Africa. Pan Afr Med J.

[4] Nguyen NT, Vu HM, Dao SD, Tran HT, Nguyen TX (2017). Digital immunization registry: evidence for the impact of mHealth on enhancing the immunization system and improving immunization coverage for children under one year old in Vietnam. Mhealth.

[5] Chandir S, Siddiqi DA, Dharma VK, Shah MT, Turab A, Khan MI, Habib A, Khan AJ (2018). Zindagi Mehfooz (Safe Life) digital immunization registry: leveraging low-cost technology to improve immunization coverage and timeliness in Pakistan. iproc.

[6] Uddin MJ, Shamsuzzaman M, Horng L, Labrique A, Vasudevan L, Zeller K, Chowdhury M, Larson CP, Bishai D, Alam N (2016). Use of mobile phones for improving vaccination coverage among children living in rural hard-to-reach areas and urban streets of Bangladesh. Vaccine.

[7] Groom H, Hopkins DP, Pabst LJ, Morgan JM, Patel M, Calonge N, Coyle R, Dombkowski K, Groom AV, Kurilo MB, Rasulnia B, Shefer A, Town C, Wortley PM, Zucker J, Community Preventive Services Task Force (2015). Immunization information systems to increase vaccination rates: a community guide systematic review. J Public Health Manag Pract.

[8] Pan American Health Organization (2017). Electronic Immunization Registry : Practical Considerations for Planning, Development, Implementation, and Evaluation.

[9] Dolan SB, Carnahan E, Shearer JC, Beylerian EN, Thompson J, Gilbert SS, Werner L, Ryman TK (2019). Redefining vaccination coverage and timeliness measures using electronic immunization registry data in low- and middle-income countries. Vaccine.

[10] Mvundura M, Di Giorgio L, Vodicka E, Kindoli R, Zulu C (2020). Assessing the incremental costs and savings of introducing electronic immunization registries and stock management systems: evidence from the better immunization data initiative in Tanzania and Zambia. Pan Afr Med J.

[11] Mvundura M, Di Giorgio L, Lymo D, Mwansa FD, Ngwegwe B, Werner L (2019). The costs of developing, deploying and maintaining electronic immunisation registries in Tanzania and Zambia. BMJ Glob Health.

[12] Gagnaire K (2020). EDIT: a tool for the greater good internet. Kati Collective.

[13] (2021). Better Immunization Data (BID) initiative. BID Learning Network.

[14] (2018). The BID initiative story: improving health services through innovation in data quality and use. Better Immunization Data (BID) Initiative.

[15] Seymour D, Werner L, Mwansa FD, Bulula N, Mwanyika H, Dube M, Taliesin B, Settle D (2019). Electronic immunization registries in Tanzania and Zambia: shaping a minimum viable product for scaled solutions. Front Public Health.

[16] Carnahan E, Ferriss E, Beylerian E, Mwansa FD, Bulula N, Lyimo D, Kalbarczyk A, Labrique AB, Werner L, Shearer JC (2020). Determinants of facility-level use of electronic immunization registries in Tanzania and Zambia: an observational analysis. Glob Health Sci Pract.

[17] Dolan SB, Alao ME, Mwansa FD, Lymo DC, Bulula N, Carnahan E, Beylerian E, Werner L, Shearer JC (2020). Perceptions of factors influencing the introduction and adoption of electronic immunization registries in Tanzania and Zambia: a mixed methods study. Implement Sci Commun.

[18] (2020). Global high resolution population denominators project. WorldPop.

[19] (2012). GADM database of global administrative areas, version 2. Global Administrative Areas (GADM).

[20] Reducing Missed Opportunities for Vaccination (MOV). World Health Organization.

[21] Wineman A, Alia DY, Anderson CL (2020). Definitions of "rural" and "urban" and understandings of economic transformation: Evidence from Tanzania. J Rural Stud.

[22] World Health Organization (2021). SCORE for health data technical package: global report on health data systems and capacity, 2020.

[23] Harrison K, Rahimi N, Danovaro-Holliday MC (2020). Factors limiting data quality in the expanded programme on immunization in low and middle-income countries: a scoping review. Vaccine.

[24] Corrêa G, Verstraete P, Soundardjee R, Shankar M, Paterson C, Hampton L, Jackson D, Muniz M, Mwamba R, Wenz K, Bratschi MW, AbouZahr C, Johnson H (2019). Immunization programmes and notifications of vital events. Bull World Health Organ.

[25] Scobie HM, Edelstein M, Nicol E, Morice A, Rahimi N, MacDonald NE, Danovaro-Holliday CM, Jawad J, SAGE Working Group on Immunization and Surveillance Data Quality and Use (2020). Improving the quality and use of immunization and surveillance data: summary report of the working group of the strategic advisory group of experts on immunization. Vaccine.

[26] Ateudjieu J, Yakum NM, Goura AP, Guenou E, Beyala LB, Amada L, Ngoche I, Kiadjieu FF, Nangue C, Djosseu EB, Kenfack B (2021). Tracking demographic movements and immunization status to improve children's access to immunization (TDM-IAI): protocol for a field-based randomized controlled trial. JMIR Res Protoc.

[27] Fiks AG, Grundmeier RW, Biggs LM, Localio AR, Alessandrini EA (2007). Impact of clinical alerts within an electronic health record on routine childhood immunization in an urban pediatric population. Pediatrics.

[28] Mayne SL, duRivage NE, Feemster KA, Localio AR, Grundmeier RW, Fiks AG (2014). Effect of decision support on missed opportunities for human papillomavirus vaccination. Am J Prev Med.

[29] Uthman OA, Sambala EZ, Adamu AA, Ndwandwe D, Wiyeh AB, Olukade T, Bishwajit G, Yaya S, Okwo-Bele J, Wiysonge CS (2018). Does it really matter where you live? A multilevel analysis of factors associated with missed opportunities for vaccination in sub-Saharan Africa. Hum Vaccin Immunother.

[30] Ndwandwe D, Uthman OA, Adamu AA, Sambala EZ, Wiyeh AB, Olukade T, Bishwajit G, Yaya S, Okwo-Bele J, Wiysonge CS (2018). Decomposing the gap in missed opportunities for vaccination between poor and non-poor in sub-Saharan Africa: a multicountry analyses. Hum Vaccin Immunother.

[31] Pancholi J, Birdie R, Guerette J, Chritz S, Sampath V, Crawford J (2020). Landscape analysis of electronic immunization registries. VillageReach.

[32] Werner L, Seymour D, Puta C, Gilbert S (2019). Three Waves of Data Use Among Health Workers: The Experience of the Better Immunization Data Initiative in Tanzania and Zambia. Glob Health Sci Pract.

[33] Pan American Health Organization (2019). Immunization Data: Evidence for Action. A Realist Review of What Works to Improve Data Use for Immunization, Evidence from Low- and Middle Income Countries.

[34] Olorunsaiye CZ, Langhamer MS, Wallace AS, Watkins ML (2017). Missed opportunities and barriers for vaccination: a descriptive analysis of private and public health facilities in four African countries. Pan Afr Med J.

[35] Ogbuanu IU, Li AJ, Anya BM, Tamadji M, Chirwa G, Chiwaya KW, Djalal ME, Cheikh D, Machekanyanga Z, Okeibunor J, Sanderson C, Mihigo R (2019). Can vaccination coverage be improved by reducing missed opportunities for vaccination? Findings from assessments in Chad and Malawi using the new WHO methodology. PLoS One.

[36] Dang H, Dao S, Carnahan E, Kawakyu N, Duong H, Nguyen T, Nguyen D, Nguyen L, Rivera M, Ngo T, Werner L, Nguyen N (2020). Determinants of scale-up from a small pilot to a national electronic immunization registry in Vietnam: qualitative evaluation. J Med Internet Res.

