# Supplementary material for: Added Value of Electronic Immunization Registries in Low- and Middle-Income Countries: Observational Case Study in Tanzania
Source: JMIR Public Health Surveill. 2022 Jan 21;8(1):e32455. doi: 10.2196/32455 (PMC8817222; doi:10.2196/32455)
Supplement: Multimedia Appendix 1 [file publichealth_v8i1e32455_app1.docx]

*Supplemental Table 1 - MOV (any vaccine) regression model full results*

| **Covariate** | **Any MOV** | | | | | |
| --- | --- | --- | --- | --- | --- | --- |
|  | **OR** | **(95% CI)** | **P value** | **aOR** | **(95% CI)** | **P value** |
| **Sex** |  |  |  |  |  |  |
| Female | Ref | - | - | Ref | - | - |
| Male | 1.00 | (0.99, 1.01) | 0.68 | 1.00 | (0.99, 1.01) | 0.90 |
| **Age** |  |  |  |  |  |  |
| 0-11 months | Ref | - | - | Ref | - | - |
| 12-23 months | 0.19 | (0.19, 0.20) | <.001 | 0.19 | (0.18, 0.19) | <.001 |
| 24-35 months | 0.25 | (0.24, 0.27) | <.001 | 0.25 | (0.23, 0.26) | <.001 |
| 36-47 months | 0.19 | (0.17, 0.22) | <.001 | 0.19 | (0.17, 0.22) | <.001 |
| 48-59 months | 0.18 | (0.15, 0.22) | <.001 | 0.18 | (0.15, 0.22) | <.001 |
| **Urbanicity** |  |  |  |  |  |  |
| Rural | Ref | - | - | Ref | - | - |
| Urban | 0.95 | (0.80, 1.12) | 0.54 | 0.90 | (0.75, 1.08) | 0.25 |
| **Ownership** |  |  |  |  |  |  |
| Private | Ref | - | - | Ref | - | - |
| Public | 1.03 | (0.90, 1.18) | 0.67 | 1.02 | (0.88, 1.18) | 0.85 |
| **Facility type** |  |  |  |  |  |  |
| Dispensary | Ref | - | - | Ref | - | - |
| Health Center | 0.92 | (0.80, 1.06) | 0.27 | 0.89 | (0.77, 1.03) | 0.11 |
| Hospital | 1.04 | (0.81, 1.33) | 0.76 | 0.99 | (0.76, 1.28) | 0.91 |
| **Facility TImR duration (at time of visit)** |  |  |  |  |  |  |
| 0-5 months | Ref | - | - | Ref | - | - |
| 6-11 months | 1.60 | (1.57, 1.62) | <.001 | 1.61 | (1.58, 1.63) | <.001 |
| 12-23 months | 2.19 | (2.15, 2.23) | <.001 | 2.27 | (2.22, 2.31) | <.001 |
| 2+ years | 3.09 | (2.97, 3.21) | <.001 | 3.15 | (3.03, 3.27) | <.001 |
