# Supplementary material for: Added Value of Electronic Immunization Registries in Low- and Middle-Income Countries: Observational Case Study in Tanzania
Source: JMIR Public Health Surveill. 2022 Jan 21;8(1):e32455. doi: 10.2196/32455 (PMC8817222; doi:10.2196/32455)
Supplement: Multimedia Appendix 2 [file publichealth_v8i1e32455_app2.docx]

*Supplemental Table 2 - MOV (OPV) regression model full results*

| **Covariate** | **OPV MOV** | | | | | |
| --- | --- | --- | --- | --- | --- | --- |
|  | **OR** | **(95% CI)** | **P value** | **aOR** | **(95% CI)** | **P value** |
| **Sex** |  |  |  |  |  |  |
| Female | Ref | - | - | Ref | - | - |
| Male | 1.00 | (0.98, 1.02) | 0.71 | 1.00 | (0.98, 1.02) | 0.88 |
| **Age** |  |  |  |  |  |  |
| 0-11 months | Ref | - | - | Ref | - | - |
| 12-23 months | 0.42 | (0.40, 0.43) | <.001 | 0.41 | (0.40, 0.43) | <.001 |
| 24-35 months | 0.32 | (0.29, 0.37) | <.001 | 0.33 | (0.29, 0.37) | <.001 |
| 36-47 months | 0.40 | (0.33, 0.49) | <.001 | 0.40 | (0.33, 0.49) | <.001 |
| 48-59 months | 0.33 | (0.24, 0.46) | <.001 | 0.33 | (0.24, 0.46) | <.001 |
| **Urbanicity** |  |  |  |  |  |  |
| Rural | Ref | - | - | Ref | - | - |
| Urban | 0.98 | (0.75, 1.27) | 0.86 | 0.96 | (0.73, 1.26) | 0.78 |
| **Ownership** |  |  |  |  |  |  |
| Private | Ref | - | - | Ref | - | - |
| Public | 0.90 | (0.72, 1.12) | 0.35 | 0.89 | (0.71, 1.12) | 0.31 |
| **Facility type** |  |  |  |  |  |  |
| Dispensary | Ref | - | - | Ref | - | - |
| Health Center | 1.17 | (0.94, 1.45) | 0.17 | 1.13 | (0.91, 1.40) | 0.28 |
| Hospital | 0.95 | (0.65, 1.40) | 0.81 | 0.92 | (0.62, 1.36) | 0.68 |
| **Facility TImR duration (at time of visit)** |  |  |  |  |  |  |
| 0-5 months | Ref | - | - | Ref | - | - |
| 6-11 months | 0.93 | (0.90, 0.95) | <.001 | 0.90 | (0.88, 0.93) | <.001 |
| 12-23 months | 0.75 | (0.72, 0.77) | <.001 | 0.73 | (0.71, 0.76) | <.001 |
| 2+ years | 0.68 | (0.63, 0.73) | <.001 | 0.67 | (0.62, 0.72) | <.001 |
