# Supplementary material for: Added Value of Electronic Immunization Registries in Low- and Middle-Income Countries: Observational Case Study in Tanzania
Source: JMIR Public Health Surveill. 2022 Jan 21;8(1):e32455. doi: 10.2196/32455 (PMC8817222; doi:10.2196/32455)
Supplement: Multimedia Appendix 3 [file publichealth_v8i1e32455_app3.docx]

*Supplemental Table 3 - Dropout (Penta) regression model full results*

| **Covariate** | **Penta dropout** | | | | | |
| --- | --- | --- | --- | --- | --- | --- |
|  | **OR** | **(95% CI)** | **P value** | **aOR** | **(95% CI)** | **P value** |
| **Sex** |  |  |  |  |  |  |
| **Female** | Ref | - | - | Ref | - | - |
| **Male** | 1.01 | (0.99, 1.03) | 0.21 | 1.02 | (1.00, 1.04) | 0.06 |
| **Age** |  |  |  |  |  |  |
| **12-23 months** | Ref | - | - | Ref | - | - |
| **24-35 months** | 0.23 | (0.22, 0.23) | <.001 | 0.23 | (0.22, 0.23) | <.001 |
| **Assigned facility urbanicity** |  |  |  |  |  |  |
| **Rural** | Ref | - | - | Ref | - | - |
| **Urban** | 0.94 | (0.80, 1.11) | 0.46 | 0.86 | (0.71, 1.04) | 0.11 |
| **Assigned facility ownership** |  |  |  |  |  |  |
| **Private** | Ref | - | - | Ref | - | - |
| **Public** | 0.99 | (0.86, 1.12) | 0.83 | 1.05 | (0.90, 1.22) | 0.57 |
| **Assigned facility type** |  |  |  |  |  |  |
| **Dispensary** | Ref | - | - | Ref | - | - |
| **Health Center** | 0.93 | (0.81, 1.07) | 0.31 | 0.95 | (0.81, 1.10) | 0.49 |
| **Hospital** | 1.23 | (0.98, 1.53) | 0.07 | 1.19 | (0.91, 1.54) | 0.20 |
| **Assigned facility stockout (% of days)** | 1.00 | (1.00, 1.01) | 0.37 | 1.00 | (1.00, 1.01) | 0.12 |
