# Supplementary material for: Added Value of Electronic Immunization Registries in Low- and Middle-Income Countries: Observational Case Study in Tanzania
Source: JMIR Public Health Surveill. 2022 Jan 21;8(1):e32455. doi: 10.2196/32455 (PMC8817222; doi:10.2196/32455)
Supplement: Multimedia Appendix 4 [file publichealth_v8i1e32455_app4.docx]

*Supplemental Table 4 - Dropout (overall) regression model full results*

| **Covariate** | **Overall dropout** | | | | | |
| --- | --- | --- | --- | --- | --- | --- |
|  | **OR** | **(95% CI)** | **P value** | **aOR** | **(95% CI)** | **P value** |
| **Sex** |  |  |  |  |  |  |
| Female | Ref | - | - | Ref | - | - |
| Male | 1.01 | (0.99, 1.02) | 0.55 | 1.01 | (0.99, 1.03) | 0.21 |
| **Age** |  |  |  |  |  |  |
| 12-23 months | Ref | - | - | Ref | - | - |
| 24-35 months | 0.19 | (0.18, 0.19) | <.001 | 0.19 | (0.19, 0.19) | <.001 |
| **Assigned facility urbanicity** |  |  |  |  |  |  |
| Rural | Ref | - | - | Ref | - | - |
| Urban | 0.87 | (0.75, 1.01) | 0.07 | 0.83 | (0.70, 0.99) | 0.03 |
| **Assigned facility ownership** |  |  |  |  |  |  |
| Private | Ref | - | - | Ref | - | - |
| Public | 1.05 | (0.94, 1.19) | 0.38 | 1.15 | (1.00, 1.33) | 0.047 |
| **Assigned facility type** |  |  |  |  |  |  |
| Dispensary | Ref | - | - | Ref | - | - |
| Health Center | 1.01 | (0.89, 1.15) | 0.84 | 1.04 | (0.91, 1.22) | 0.58 |
| Hospital | 1.24 | (1.01, 1.52) | 0.04 | 1.27 | (1.00, 1.61) | 0.06 |
| **Assigned facility stockout (% of days)** | 1.00 | (1.00, 1.00) | 0.70 | 1.00 | (1.00, 1.01) | 0.28 |
